# Does environmental public interest litigation improve the urban land green use efficiency?—Evidence from a quasi-natural experiment in China

**DOI:** 10.1371/journal.pone.0303850

**Published:** 2024-05-23

**Authors:** Ling Zhao, Can Xie, Hao Huang

**Affiliations:** 1 School of economics, Xihua University, Chengdu, Sichuan, China; 2 School of Public Finance and Taxation, Southwestern University of Finance and Economics, Chengdu, Sichuan, China; National University of Sciences and Technology, PAKISTAN

## Abstract

Environmental public interest litigation (EPIL) is a significant part of the judicial system; it is aimed at strengthening judicial protections and safeguarding public interests. Based on the quasi-natural experimental setting of China’s EPIL pilot project, this study examines the impact of EPIL on the country’s urban land green use efficiency (ULGUE). The findings show that effectively implementing EPIL enhances ULGUE. Specifically, this policy has led to a 6.6% increase in ULGUE in pilot cities, and its impact has grown stronger over time. Mechanism analysis results show that EPIL mainly enhances ULGUE by strengthening environmental supervision and law enforcement, by increasing public participation in environmental governance, and by promoting green innovation and industrial structure upgrades. Furthermore, heterogeneity analysis revealed that the positive effects of this policy implementation are more pronounced in resource-based cities, cities with open environmental information, and cities with high marketization. This paper provides empirical evidence for the effectiveness of environmental governance via EPIL.

## 1. Introduction

According to the State of the World’s Cities 2022 Report released by UN-Habitat, more than half of the world’s population lives in cities. By 2050, close to 70% of the total global population is expected to be urban population. China, one of the fastest urbanizing countries in the world, reached an urbanization rate of 65.22% in 2022. Urban land resources play a vital role in urban development and ecological preservation. However, with continuous urbanization worldwide, these resources are increasingly under strain, and the prevalent land use practices face significant challenges [1,2]. Protecting urban land resources and achieving rational and sustainable green utilization become crucial for urban green transformation, low-carbon development, and overall economic and social progress. Improving urban land green use efficiency (ULGUE) has become an important aspect of national and regional governmental efforts to develop cities sustainably.

In general, the rule of law ensures the effective enforcement of environmental regulations [3], providing the backbone that supports sustainable, green urban development. Over the years, China has actively undertaken environmental judicial reforms, establishing an environmental public interest litigation (EPIL) system to address the absence of public interest protection subjects. This system empowers procuratorial organs to supervise the legal enforcement of environmental protection duties. An innovative judicial system, EPIL plays a crucial role in safeguarding social and public interests [4].

The EPIL system has been implemented since 2015 as a pilot program in 13 regions in China, including Beijing and Inner Mongolia, its primary focus lies in cases involving ecological environment and resource protection. But there is still limited literature on its practical effects. Land as a resource is vital for supporting urban development, and a city’s ULGUE to a certain extent reflects the performance of its environmental governance [5,6]. With that in mind, in this paper, we examine the environmental governance performance of EPIL from the perspective of ULGUE.

Following Tone and Zhang, we used a super-SBM model to construct an ULGUE index and examined the impacts of EPIL based on panel data from prefecture-level cities in China from 2010 to 2020 [7,8]. We determined that implementing EPIL effectively enhances a city’s ULGUE. Specifically, this policy has led to a 6.6% increase in ULGUE in pilot cities, and its impact has grown stronger over time. Through cross-section analysis, we determined that the positive effects are more pronounced in resource-based cities, cities with open environmental information, and cities with high marketization. Mechanism analysis revealed that EPIL mainly enhances ULGUE by strengthening environmental supervision and law enforcement, increasing public participation in environmental governance, and promoting green innovation and industrial structure upgrades.

The main contributions of this paper are as follows: First, it enriches the research on improving ULGUE by enhancing policy and institutional design. In recent years, temperatures globally have hit record highs and extreme weather phenomena have occurred frequently, prompting governments around the world to explore low-carbon development [9,10]. ULGUE has since become a hot topic. Previous researchers have mainly focused on the impact of command-type, weak-constraint-type, and market-transaction-type environmental policies on ULGUE [11–13], but there is limited literature regarding the role of environmental justice system reform in promoting this efficiency. With this paper, we attempt to fill this gap.

Second, we empirically tested the impact of the EPIL pilot on ULGUE to provide theoretical support for whether this system can improve urban environmental governance performance. Because of the many factors affecting the legal system and environmental governance performance, endogeneity is often a problem in the empirical study of the relationship between the two [14]. For this paper, we used the unique setting of China’s judicial system reform pilot to identify the causal relationship between EPIL and environmental governance performance; we offer a useful reference for subsequent literature on legal systems and environmental protection.

Third, we analyzed the mechanism of how EPIL improved ULGUE from multiple dimensions, such as environmental law enforcement supervision and public participation awareness, which enables opening up the black box of the impact of judicial system reform on environmental governance. We further analyze the heterogeneity of this policy effect according to differing urban characteristics to serve as a useful reference for using the legal system to improve ULGUE under different economic development conditions, especially in emerging economies.

The remainder of this study proceeds as follows. Section 2 describes the policy background and hypothesis development. Section 3 presents the methodology, variables, and data. Section 4 reports the empirical results and robustness tests, and section 5 provides further analysis for mechanism validation and heterogeneity analysis. The conclusions are presented in section 6.

## 2. Institutional background and hypothesis development

### 2.1 Institutional background

#### 2.1.1 Establishing China’s EPIL system

In the field of environmental law, the establishment of the EPIL system in China has been a significant topic of discussion. This process began in 2005, when the “Decision of the State Council on Implementing the Scientific Development Outlook and Strengthening Environmental Protection” first proposed EPIL. However, it got elevated to the legislative level and made a tangible impact over several years [4].

A milestone was reached in 2007 with the establishment of the Ecological Protection Court at Qingzhen City People’s Court in Guizhou Province, which marked the country’s first environmental court and represented an initial foray into EPIL. Subsequently, in 2014, the Central Committee of the Communist Party of China proposed exploring a system for procuratorial organs to initiate public interest litigation. In the same year, the Supreme People’s Court established the Environmental Resources Trial Division, officially establishing the environmental court system [15].

Building on these developments, in 2015, the Standing Committee of the National People’s Congress passed the “Decision on Authorizing the Supreme People’s Procuratorate to Carry out Pilot Programs of Public Interest Litigation in Some Regions.” This decision mandated a two-year pilot program of public interest litigation in select cities across 13 provinces, with a focus on ecological environment and resource protection. According to statistics from the Supreme People’s Procuratorate, during the pilot period, procuratorial organs handled a total of 9,053 public interest litigation cases.

Among these cases, 6,527 were related to public interest litigation in the field of ecological environment and resource protection. The procuratorial organs supervised the restoration of 12.9 hectares of polluted or destroyed land, as well as over 180 square kilometers of polluted water sources. Additionally, they urged more than 1,700 illegal enterprises to rectify their actions. Through EPIL, more than 8.9 billion yuan of direct economic losses were recovered.

#### 2.1.2 Working logic of China’s EPIL system

The concept of EPIL originated in developed countries and was gradually adopted and modified by emerging economies [16]. In China, EPIL is primarily initiated by procuratorial organs, resulting in a judicial system with distinct Chinese characteristics [17]. EPIL aims to safeguard social public interests and strengthen legal supervision via administrative and civil environmental public interest litigation.

The administrative litigation is governed by the procuratorial organ. If the local procuratorate finds that an administrative organ has illegally exercised its powers or failed to act and has thereby infringed on the country’s interests, it can make procuratorial suggestions to the administrative organ and file litigation with the people’s court. EPIL encourages administrative agencies to use the law to protect the ecological interests of the public [18].

For the civil public interest litigation, the procuratorial organs and social organizations that meet legal requirements have the right to initiate the litigation and correct or stop natural or legal persons from polluting the environment and damaging social public interests [19]. EPIL fundamentally differs from traditional individual rights protection litigation. It focuses on safeguarding environmental rights and interests, placing greater emphasis on maintaining objective legal order and realizing social public interests [20]. Therefore, this system’s effective operation relies on the coordinated cooperation among the legal environment, policy participation, and supervision mechanisms.

### 2.2 Hypothesis development

Land supports social development; its uses reflect an economy’s production scale and technological efficiency, and it determines the material environmental conditions of human life [21]. In recent years, it has been widely recognized that urban land use should not be limited to pursuing economic benefits but should include the environmental benefits generated by land use [8]. Green development and environmental governance cannot be separated from legal protection [22].

In the dynamic process of land resource development and utilization, the legal enforcement of environmental regulations is undoubtedly critical for ensuring the green development of land resources. However, because of the pressure of economic growth, the enforcement of government environmental regulations is often challenged. Thus, a significant mission of EPIL is to facilitate the monitoring and enforcement of regulations that are already technically in effect.

Urban residents are the most direct beneficiaries of ULGUE improvements as well as being key providers of information about problematic land use incidents. Another important facet of EPIL is awakening people’s awareness of the need to participate by reporting incidents and by participating in public interest litigation when invited, which will avoid needless public land destruction and strengthen the effect of EPIL. The main body of land development is micro-enterprises, and the internal driving force to improve ULGUE is to remove the economic benefits from committing environmental violations and increase penalties. The aim is to encourage enterprises to carry out more green innovation and ultimately promote structural transformation.

For the purposes we described above, with this study, we analyzed the effect path of EPIL on ULGUE from the aspects of law enforcement supervision, public participation, and endogenous motivation of enterprises in land resource development and utilization. First, in many regions globally, especially in developing countries, because of the pressure of economic growth, the legal enforcement of government environmental regulations has been ineffective [23]. As we described, countries focus on improving economic performance, and land development is often at the cost of the ecological environment. Therefore, it is necessary to strengthen the enforcement of existing environmental regulations, and EPIL gives local administrative departments the legal backing to effectively fulfill their environmental governance responsibilities. In the event of violations, procuratorial organs can propose prelitigation inspection suggestions or file public interest litigation to administrative organs in accordance with the law [19]. Therefore, EPIL can encourage local governments to strictly implement central environmental policies and promptly correct environmental damage issues resulting from land use.

Second, the green utilization and development of land resources also depend on the public’s participation and robust external constraint mechanisms. Improving ULGUE requires not only effective coordination between environmental administration and environmental justice but also the public’s active involvement and shared responsibility. People’s environmental demands also have an impact on environmental pollution control as a form of informal environmental regulation [24].

The trial implementation of the EPIL system enables the public to participate in regional environmental pollution control by making complaints and visits and by learning about environmental pollution. The opening of the National Prosecutorial 12309 website has expanded the channels for the public to respond to environmental demands. The joint participation of the public in environmental pollution control can increase the numbers of cases that procuratorial organs are aware of and can adjudicate, including providing evidence that can be difficult to collect for EPIL cases. EPIL helps reflect the public’s environmental interests and demands of the public and increase their confidence in and enthusiasm for maintaining public interests and participating in environmental governance [25].

Third, EPIL is important for promoting green technology innovation and facilitate the transformation and upgrading of the industrial structure. Prior research has established that strict environmental laws can effectively constrain corporate behavior [26,27]. Companies comply with environmental regulatory requirements based on cost–benefit analyses of the results of not complying [28]. When it becomes expensive to violate strong environmental regulations, company leaders often take more proactive environmental governance strategies, such as carrying out greater green technology innovation activities, to avoid significant economic losses and increase the probability of receiving “innovation compensation” benefits [29]. The EPIL system will encourage enterprises to invest in environmental protection technology and transform from end-of-pipe pollution control to holistic green technology innovation [30].

Moreover, the EPIL has significant implications for industrial structure. On the one hand, it incentivizes local governments to employ various policy instruments such as finance, taxation, and administrative penalties to adjust the industrial structure [31]. This includes relocating high-pollution enterprises that are unable to transform because of cost. Consequently, it creates space for more advanced industries with higher value-added products and stricter emission standards [32]. This transformation of the industrial structure not only increases the green development of land resources but also optimizes the spatial distribution of industries. This, in turn, promotes efficient circulation and intensive utilization of elements required for green development, thereby collectively improving the green utilization efficiency of urban land [33,34]. Based on the above three aspects, we hypothesize that EPIL and ULGUE are positively correlated.

## 3. Research design

### 3.1 Model specification

The EPIL system was piloted in 13 regions of China, including Beijing, Jiangsu, and Fujian; it started in 2015. It was developed following examinations of public data and information from the judgment documents network. Ultimately, Beijing, and 80 prefecture-level cities under the jurisdiction of the pilot provinces were selected as the pilot areas, and the unselected cities in the pilot provinces served as the control group. Table 1 presents the specific list of treatment groups.

**Table 1:** Pilot regions for EPIL.

Pilot time	Pilot city
**The year 2015**	Baisha, Yantai, Qingdao, Guangzhou, Zhanjiang, Zhangye, Nanping
**The year 2016**	Chifeng, Beijing, Tonghua, Dezhou, Xuzhou, Taizhou, Yancheng, Shiyan,
	Jingzhou, Sanming, Guiyang
**The year 2017**	Baotou, Hulun Buir, Hohhot, Ordos, Ulanqab, Jilin,
	Siping, Baicheng, Changchun, Nanjing, Changzhou, Suzhou, Wuxi, Yangzhou,
	Lu ’an, Hefei, Xuancheng, Su’zhou, Chuzhou, Wuhu, Bengbu, Fuyang,
	Bozhou, Quanzhou, Fuzhou, Longyan, Xiamen, Weifang, Liaocheng, Linyi,
	Xianning, Yichang, Wuhan, Huanggang, Huangshi, Xiangyang, Shantou, Shenzhen,
	Qingyuan, Zhaoqing, Liupanshui, Bijie, Zunyi, Anshun, Lincang, Kunming,
	Pu ’er, Qujing, Baoshan, Xianyang, Baoji, Yulin, Hanzhong, Weinan,
	Xi ’an, Ankang, Lanzhou, Tianshui, Qingyang, Baiyin, Jiuquan, Longnan, Dingxi.

Because EPIL was implemented at different times in the different provinces, we analyzed its impacts on ULGUE following staggered difference-in-differences method (DID) as the benchmark identification strategy [35] using Eq (1).

$$ULGUE_{it}=\alpha_{0}+\alpha_{1}policy_{it}+\sum_{j}\alpha_{j}\times control+\gamma_{i}+\theta_{t}+\varepsilon_{it}$$ (1)

where *ULGUE*_*it*_ represents the urban land green utilization efficiency and *i* and *t* denote the *i*-th city and the *t-*th year, respectively. The EPIL system is represented by *policy*_*it*_ *= treat*_*i*_ * post_*it*_, the primary explanatory variable in this study. The coefficient of *policy*_*it*_ reflects the effectiveness of the implemented policy. The variable *treat*_*i*_ is the policy grouping variable, with *treat*_*i*_ = 1 indicating the treatment group and *treat*_*i*_ = 0 indicating the control group. The variable *post*_*it*_ is the policy timing variable, indicating whether city *i* implemented the EPIL policy in period *t*. It takes the value of 0 before and 1 after policy implementation. The variable control represents other control variables that could influence ULGUE. The parameters *γ*_*i*_ and *θ*_*t*_ represent city and time fixed effects, respectively. The error term *ε*_*it*_ denotes robust standard errors clustered at the city level.

### 3.2 Variable definition

#### 3.2.1 Dependent variable

ULGUE refers to the efficiency of land space utilization, including ecological and environmental losses during land use. It expands upon traditional land use efficiency measurements by incorporating undesirable outputs into the assessment model. The super-efficiency SBM model, as discussed in the existing literature, calculates system efficiency scores by quantifying and weighting the input and output indicators based on the Data Envelopment Analysis (DEA) model [36].

By integrating the advantages of the super-efficiency and SBM models, this approach addresses the practical challenge of traditional SBM-undesirable models, where the efficiency value of effective decision-making units (DMUs; in each city in the sample) can no longer be decomposed, leading to a loss of relevant information [37]. The non-oriented super-efficiency SBM model allowed us to include unexpected outputs in gauging the green utilization efficiency of urban land in the pilot cities. This approach is inspired by Tone’s work in the field [38]. The model construction is as follows:

$$\rho =\min\frac{1-\frac{1}{n}\sum_{1}^{n}\frac{s_{i}^{-}}{x_{io}}}{1+\frac{1}{s_{1}+s_{2}}(\sum_{r=1}^{s_{1}}\frac{s_{r}^{g}}{Y_{r_{o}}^{g}}+\sum_{r=1}^{s_{2}}\frac{s_{r}^{b}}{Y_{r_{o}}^{b}})}$$ (2)

$$\begin{array}{l} s.t.x_{o}=X\lambda +S^{-}\\ \;y_{o}^{g}=Y^{g}\lambda -S^{g}\\ \;y_{o}^{b}=Y^{b}\lambda -S^{b}\\ \;S^{-}\geq 0,S^{g}\geq 0,S^{b}\geq 0,\lambda \geq 0 \end{array}$$ (3)

In Eq (2), *ρ* represents ULGUE, *n* represents the types of input ($x=[x_{1},x_{2},\ldots ,x_{n}]\in R^{m\times n}$) of m DMUs, *S*_*1*_ represents the expected output ($Y^{g}=[y_{1}^{g},..,y_{m}^{g}]\in R^{s_{1}\times m}$), and *S*_*2*_ represents the desired output ($Y^{b}=[y_{1}^{b},..,y_{m}^{b}]\in R^{s_{2}\times m}$). In Eq (3), *S*^*−*^represents the redundancy of input relative to the optimal input, *S*^*b*^ represents the redundancy of non-desired output relative to the efficiency of non-desired output, *S*^*g*^ represents the insufficiency of expected output relative to the efficiency of expected output, *r* represents the *r*-th DMU, *r*_0_ represents the DMU to be evaluated, and*λ*is the weight vector. Because ULGUE emphasizes the input–output efficiency that can be supported per unit area of land, we selected per unit area as the input and output for the evaluation index: labor input per unit area, fixed asset investment per unit area, energy consumption per unit area, and the completed amount of investment in industrial pollution control per unit area are selected as input indicators. We selected per unit value-added of secondary and tertiary industries as the main expected output.

Furthermore, we took per capita disposable income of urban residents as the social benefit indicator and included green coverage rate as the environmental output indicator for comprehensive calculation. Finally, we input the emissions of industrial wastewater, industrial sulfur dioxide, and industrial dust per unit area as the non-desired output indicators. We constructed the pollution index using entropy weight and then incorporated that into the evaluation model to eliminate the dimensional difference. The specific index construction system is shown in Table 2.

**Table 2:** The indicator system for ULGUE.

Classes	Variable description
**Input**	*Average capital factor input for land utilization*: Urban fixed asset investment in city districts (in hundred million yuan), taking 2010 as the base period, using the fixed asset investment price index and converting the nominal fixed asset investment value into comparable real fixed asset investment. The perpetual inventory method is used to calculate the capital stock of each city over the years, which is then divided by the urban construction land area in city districts (in square kilometers). *Average labor factor input for land utilization*: The total number of urban employed persons, including urban units and urban private workers (in ten thousand people), divided by the urban construction land area in city districts (in square kilometers). *Average Energy Consumption for land utilization*: Energy consumption converted into standard coal equivalent (in ten thousand tons) divided by the urban construction land area in city districts (in square kilometers). *Average Industrial Pollution Control Investment for land utilization*: The completed investment in industrial pollution control (in ten thousand yuan) divided by the urban construction land area in city districts (in square kilometers).
**Desirable Output**	*The added value of the secondary and tertiary industries in urban city districts* (in hundred million yuan). To enhance data comparability, the nominal value of industrial added value is converted into comparable real added value using the GDP index, with 2010 as the base year. *Per Capita Disposable Income of Urban Residents* (in yuan). *Green coverage rate in the built-up area* (%).
**Undesirable Output**	*The selection of industrial wastewater discharge (in ten thousand tons)*, *industrial sulfur dioxide emissions (in tons)*, *and industrial soot emissions (in tons) as the three main sources of urban pollution as the undesirable outputs*. Due to the requirement of the DEA model for a limited number of outputs, and to eliminate the disparity in scales caused by the different measurement units of the three undesirable outputs, the comprehensive index of the undesirable outputs is calculated using the entropy weighting method.

#### 3.2.2 Environmental public interest litigation

EPIL was the explanatory variable in this study (*policy*_*it*_). Specifically, *policy*_*it*_ *= treat*_*i*_ * post_*it*_ represents the EPIL system implementation. The variable *treat*_*i*_ is the policy grouping variable, with *treat*_*i*_ = 1 indicating the treatment group and *treat*_*i*_ = 0 indicating the control group. The variable *post*_*it*_ is the policy timing variable, indicating whether city *i* implemented the EPIL policy in period *t*. It takes the value of 0 before policy implementation and 1 after policy implementation.

In addition, environmental policy assessment often faces endogeneity that can interfere with identifying policy effectiveness. However, we believe that the endogeneity selectivity of China’s EPIL pilot policy is relatively weak. Because the EPIL pilot program is determined by the Supreme People’s Procuratorate authorized by the National People’s Congress of China and is exogenous to the city itself. Furthermore, from the list of pilot cities in Table 1 above, it can be seen that the determination of pilot cities in different regions, including the eastern, central, and western regions, with different levels of economic development and pollution conditions, generally presents uniform and random characteristics.

#### 3.2.3 Mediating variables

Existing literature indicates that environmental enforcement supervision (*lnEES*) is a horizontal supervision that improves the strength and efficiency of environmental law enforcement [18], thus enhancing the efficiency of environmental protection. Therefore, we consider the environmental law enforcement efforts a potential mechanism and use the number of environmental administrative penalty cases in each city as the proxy variable. Improving ULGUE requires not only effective coordination between environmental administration and justice but also the active involvement and shared responsibility of the public. Public participation in environmental governance (*PPEG*) as an informal environmental regulation could encourage people to participate in environmental governance, and once their demands are met, it would tremendously inspire their enthusiasm. Accordingly, we chose the total number of proposals from local People’s Congresses and Political Consultative Conferences to reflect the level of public participation.

Additionally, EPIL has imposed extensive environmental constraints, and local governments are being forced to promote green technology innovation and accordingly transform and upgrade traditional industries. In this study, we calculated the industrial structure upgrade (*ISU*) as the ratio of value added by the tertiary industry to that of the secondary industry because the more developed the secondary industry, particularly if it is heavy, the more serious the environmental pollution will be. In contrast, tertiary industries rely less on energy inputs and thereby reduce emissions [24,39]. We defined green technological innovation (*lnGTI*) according to Du et al. (2021) to represent the logarithm of the sum of the number of green invention applications and the number of green utility model patent applications [31].

#### 3.2.4 Control variables

To reduce the influence of other factors on the impacts of EPIL on ULGUE, we input several control variables based on relevant literature: economic development level (*GDP*), measured by GDP growth rate and its squared term; urbanization level (*UR*), measured as the ratio of urban population to total population; research and development level (*LRD*), measured as the proportion of scientific and technological expenditures to GDP; foreign investment level (*FII*), measured as the proportion of total foreign direct investment to GDP; and the availability of urban resources (*URE*), which we measured as the proportion of mining workers to total employment. We give the specific definitions of these variables in Table 3.

**Table 3:** Variable definition.

Variable	Definition	Variable description
***ULGUE***	Urban Land Green Utilization Efficiency	The specific calculation method for efficiency value is described in the previous section.
***policy***_***it***_	EPIL Policy	The specific assignment method for policy variables is described in the previous section.
***lnEES***	Environmental Enforcement Supervision	The natural logarithm of the number of environmental administrative penalty cases in each city.
***PPEG***	Public Participation in Environmental Governance	The total number of proposals submitted by the people’s congress and the political consultative conference in the region.
***ISU***	Industrial Structure Upgrading	The ratio of the value added of the tertiary industry to the value added of the secondary industry.
***lnGTI***	Green Technology Innovation	The natural logarithm of the sum of the number of green invention applications and the number of green utility model patents granted in the current year.
***GDP***	Level of Economic Development	The GDP growth rate.
***GDP*** ^***2***^	Square of GDP	The square term of GDP
***UR***	Urbanization Rate	The proportion of urban population to the total population.
***LRD***	Level of Research and Development	The proportion of scientific and technological expenditure to GDP.
***FII***	Foreign Investment Intensity	The ratio of total foreign direct investment to GDP.
***URE***	Urban Resource Endowment	The proportion of mining workers to the total employed population.

### 3.3 Sample selection and data sources

We selected data from 283 cities at the prefecture level and above in China from 2010 to 2020, specifically, original data from China Statistical Yearbook, the China Urban Statistical Yearbook, and the China Environmental Yearbook. To eliminate the impact of price factors, we input cities’ 2010 GDPs; we also converted the foreign investment amounts in USD to RMB based on the average exchange rate over the years (S1 Data).

We took the green patent data from the cities and different enterprises from the China Research Data Service Platform, which filters and matches the patent data of the China National Intellectual Property Administration according to the green patent standards of the World Intellectual Property Organization. We compiled data on different cities’ environmental administrative punishment cases from the case documents of Peking University’s magic weapon. To minimize the bias in the results caused by outliers, we winsorized the main variables at a trimming level of 0.5%.

### 3.4 Descriptive statistics

Table 4 gives the descriptive statistics of the main variables; higher *ULGUE* indicates higher urban land green use efficiency. The mean and median *ULGUE* in the control group were 0.329 and 0.264, respectively, which are lower than those in the treatment group, 0.422 and 0.323. The *lnEES* means were 1.075 and 1.267, respectively, indicating that the average number of environmental administrative penalty cases in the non-pilot areas during the sample period was approximately 2.93 against 3.90 in the pilot areas. Among the control variables, mean *GDP* as an example was 0.084 and 0.092, respectively, for the control and treatment groups. Table 4 shows that there were no outliers in any of the variables’ statistical indicators, but we still need further verification to determine whether the difference in *ULGUE* between pilot and non-pilot areas was caused by the EPIL policy.

**Table 4:** Descriptive statistics for variable grouping.

*VARIABLES*	Control group						Treatment group					
	N	mean	sd	min	p50	max	N	mean	sd	min	p50	max
***ULGUE***	2,222	0.329	0.212	0.116	0.264	1.081	891	0.422	0.267	0.116	0.323	1.081
***lnEES***	2,222	1.075	4.240	-12.27	2.265	7.359	891	1.267	3.568	-10.780	1.680	7.359
***PPEG***	2,222	5.821	7.005	0.0403	3.607	37.421	891	5.916	5.947	0.040	3.857	37.422
***lnGTI***	2,222	4.966	1.573	1.386	4.860	9.296	891	5.401	1.891	1.386	5.398	9.296
***ISU***	2,222	1.282	0.594	0.264	1.177	3.514	891	1.154	0.485	0.264	1.102	3.514
***GDP***	2,222	0.084	0.042	-0.047	0.081	0.181	891	0.092	0.041	-0.047	0.089	0.181
***GDP*** ^***2***^	2,222	0.890	0.712	0.006	0.672	3.312	891	1.017	0.745	0.006	0.792	3.312
***UR***	2,222	0.386	0.215	0.081	0.330	1.243	891	0.414	0.256	0.102	0.345	1.243
***LRD***	2,222	0.015	0.014	0.001	0.011	0.079	891	0.019	0.018	0.001	0.013	0.079
***FII***	2,222	0.016	0.0165	-0.005	0.012	0.074	891	0.016	0.017	-0.005	0.011	0.074
***URE***	2,222	0.057	0.0935	-0.001	0.013	0.405	891	0.032	0.054	-0.001	0.001	0.374

## 4. Empirical results and analysis

### 4.1 Results of the primary tests

Table 5 reports the results for Eq (1). Column (1) is the univariate regression result, Column (2) is the result of adding time and city fixed effects, and Column (3) is the result of adding control variables. Consistently across columns (1) to (3), the coefficients of *Policy* are significantly positive (two-tailed p < 0.01), indicating that ULGUE increased significantly following the enactment of EPIL policy. The results are economically significant. For example, in Column (3), the coefficient on *Policy* is 0.066, indicating that ULGUE was 6.6% higher in areas affected by EPIL than in the non-affected areas.

**Table 5:** Primary regression results.

*VARIABLES*	(1)	(2)	(3)	(4)
***policy***_***it***_	0.197***	0.071***	0.066***	0.065***
	(12.42)	(5.00)	(2.76)	(2.74)
***GDP***			0.933***	0.941***
			(4.23)	(4.21)
***GDP*** ^***2***^			-0.051***	-0.050***
			(-3.65)	(-3.54)
***UR***			-0.133	-0.134
			(-1.28)	(-1.28)
***LRD***			1.725**	1.706**
			(2.42)	(2.39)
***FII***			0.432	0.426
			(0.97)	(0.95)
***URE***			0.418**	0.420**
			(2.31)	(2.32)
***Constant***	0.333***	0.753***	0.312***	0.311***
	(81.89)	(12.36)	(6.94)	(6.85)
**City FE**	NO	YES	YES	YES
**Year FE**	NO	YES	YES	YES
**Observations**	3,113	3,113	3,113	3,098
**Adj-R** ^***2***^	0.075	0.638	0.647	0.647

Robust t-statistics in parentheses.

*** p<0.01

** p<0.05

* p<0.1.

It is worth pointing out that, due to the scale and depth of intervention is subject to civil society response to the legal statutes and their dedication to pursue litigation which can vary significantly across jurisdictions, the estimated results of the DID model may be inaccurate and difficult to explain. Based on this, we employed propensity score matching (PSM) and selected the GDP growth rate and regional legalization index as key indicators. Through 1:1 nearest neighbor matching with replacement, we assembled a control group that closely resembled the experimental group, facilitating a regression analysis based on Eq (1). The regression outcomes are presented in Column (4), Table 1. The magnitude and significance of the policy coefficient (0.065, P value< 0.01) were comparable with those obtained in Column (3), Table 1, thereby affirming the credibility of the benchmark regression results presented in this paper.

In addition, from the perspective of economic significance, the 6.6% increase in ULGUE following the implementation of EPIL exceeds the findings from the existing literature on the impact of policies such as low-carbon city construction (2.58%) and free trade zone construction (5.71%), reflecting a positive impact of EPIL policy on ULGUE [36,40]. Increasingly, public environmental concern and related judicial system reforms in developing countries are playing a key role in environmental pollution control [28,41–43]. Indeed, as per the official summary of the EPIL pilot work by the Supreme People’s Procuratorate of China, as of June 2017, the procuratorial organs in the pilot areas had handled 6,527 public interest litigation cases in ecological environment and resource protection; mandated restoring 129,000 hectares of polluted and damaged farmland, forest land, wetlands, and grasslands and more than 180 square kilometers of polluted water sources; and required more than 1,700 illegal enterprises to make rectifications. This provides further support for the conclusion that the EPIL system is effectively improving ULGUE around China.

### 4.2 Parallel trends test

It was deemed necessary to conduct a parallel trend test before using the DID model. The parallel trend test examines whether there are consistent trends in the changes of ULGUE between pilot and non-pilot cities prior to the implementation of EPIL policy. This analysis helps ensure that the benchmark regression results are attributable to the EPIL policy rather than time trend changes between the two city groups. Following Zhou et al., an event study approach was employed to conduct the parallel trend test [44].

**Fig 1:**
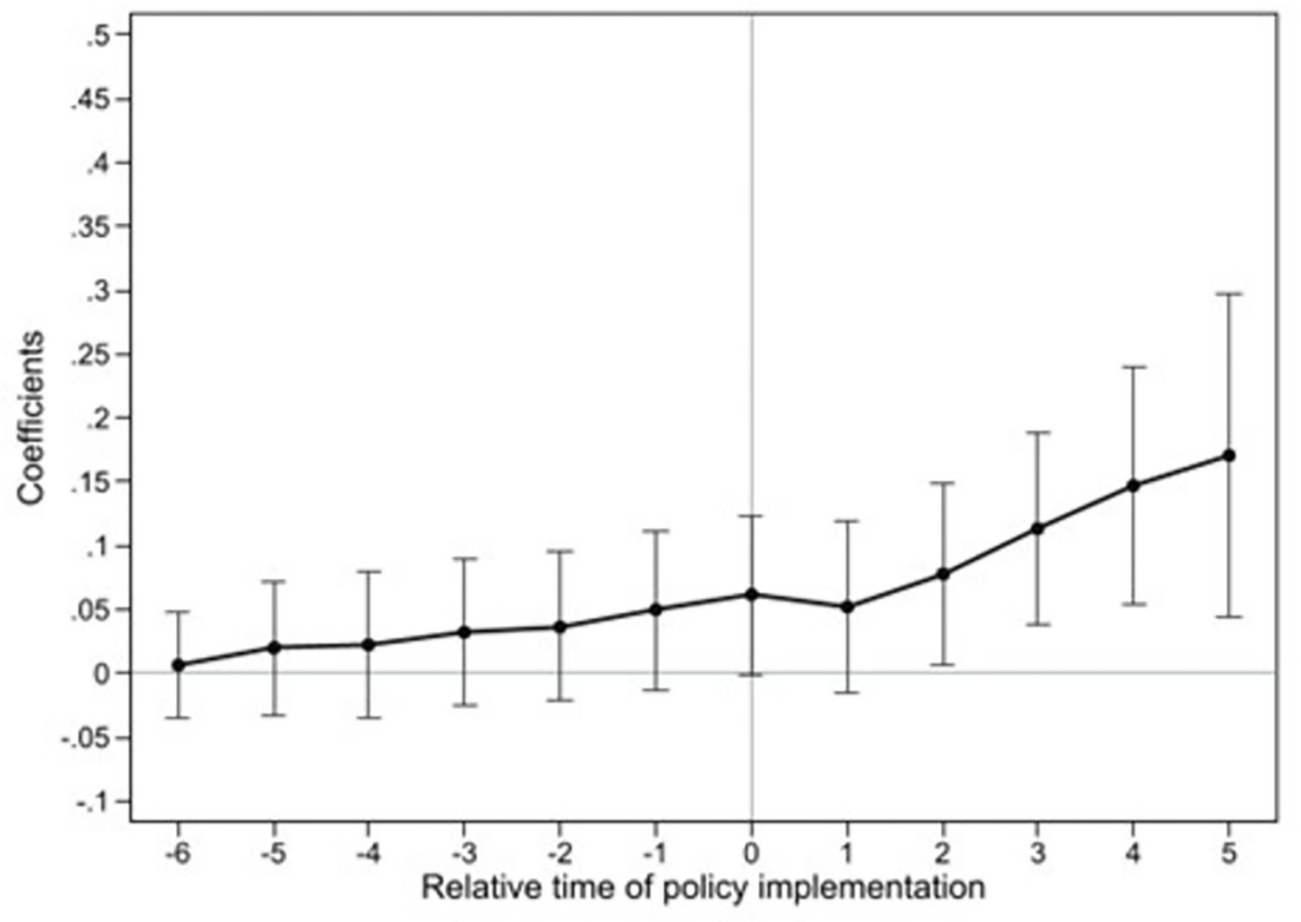
Parallel trend test results.

Fig 1 graphically displays the results of the parallel trend test. The figure shows that the regression coefficients are not significant before the policy implementation, indicating no systematic difference in ULGUE between the pilot and non-pilot cities. This validates the parallel trend assumption. Therefore, the baseline regression results are not influenced by inherent time trends between the two groups.

Moreover, after the second period of the pilot, the coefficient becomes significantly positive, and its absolute value keeps increasing, indicating that the promotion effect of the EPIL pilot on ULGUE strengthens over time.

### 4.3 Robustness test

#### 4.3.1 Placebo-controlled trial for pilot time

To avoid the potential differences in ULGUE caused by variations in time across cities, we studied the pilot across two periods, 2014–2017 and 2013–2017. We thereby constructed two virtual explanatory variables, *policy2*_*it*_ and *policy3*_*it*_, for the regression analysis. The regression results, shown in Columns (2) and (4) of Table 6, indicate that the coefficients of the main explanatory variables are not statistically significant after we controlled for other variables. This suggests that there is no systematic difference in ULGUE between pilot and non-pilot cities over time, further confirming the robustness of the benchmark regression results.

**Table 6:** Placebo test.

*VARIABLES*	(1)	(2)	(3)	(4)
***policy2***_***it***_	-0.004	-0.016		
	(-0.29)	(-1.05)		
***Policy3***_***it***_			-0.016	-0.025
			(-0.92)	(-1.42)
***GDP***		0.921***		0.938***
		(4.24)		(4.33)
***GDP*** ^***2***^		-0.055***		-0.054***
		(-3.90)		(-3.85)
***UR***		-0.134		-0.134
		(-1.21)		(-1.21)
***LRD***		1.906***		1.917***
		(2.65)		(2.67)
***FII***		0.466		0.471
		(1.03)		(1.04)
***URE***		0.454**		0.452**
		(2.51)		(2.52)
***Constant***	0.356***	0.321***	0.358***	0.320***
	(227.21)	(6.78)	(154.18)	(6.80)
**City FE**	YES	YES	YES	YES
**Year FE**	YES	YES	YES	YES
**Observations**	3,113	3,113	3,113	3,113
**Adj-R** ^***2***^	0.633	0.643	0.634	0.644

Robust t-statistics in parentheses.

*** p<0.01

** p<0.05

* p<0.1.

#### 4.3.2 Placebo-controlled trial for pilot cities

**Fig 2:**
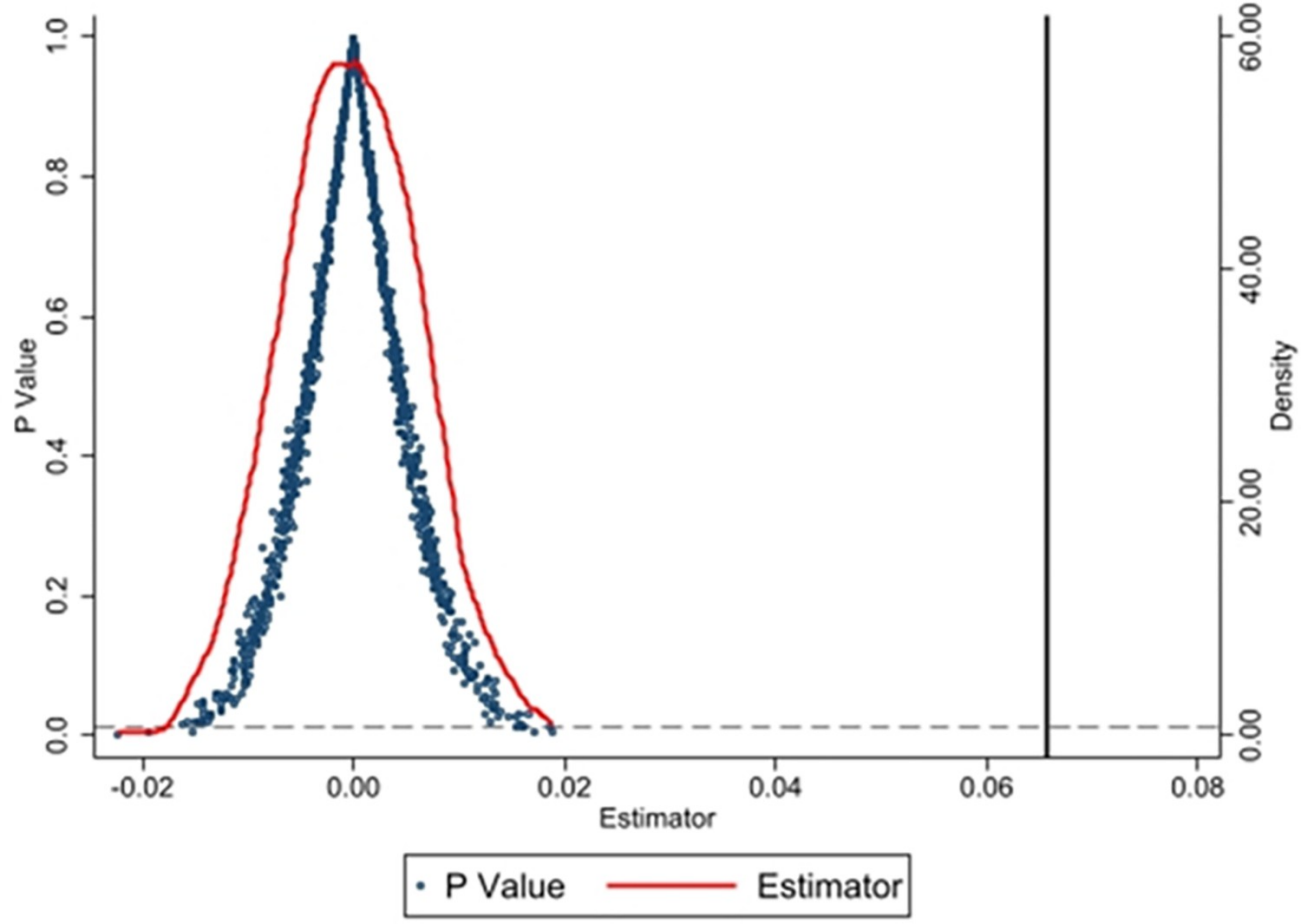
Placebo-controlled trial results.

To further demonstrate that the improvement in ULGUE indeed derives from the EPIL system and to mitigate the influence of other unobservable factors on the empirical results, we conducted a placebo test following Lu et al. [45]. Specifically, we took a random selection from the 283 prefecture-level cities as the treatment group and then re-estimated the staggered DID Eq (1) to simulate the estimate of the coefficient representing the impact of the EPIL system on ULGUE. We performed this process 1,000 times, generating 1,000 regression coefficients and their corresponding p values for the core explanatory variable. We next created a kernel density plot and a combination plot of the p values, as illustrated in Fig 2.

This figure shows that the coefficients derived from the random samples conform to a normal distribution and are centered around 0. The majority of the regression coefficients possess p values greater than 0.01. The black solid line represents the estimated coefficient of the benchmark regression model mentioned earlier, which significantly differs from the mean of the kernel density distribution. Consequently, we can conclude that the policy effect of the EPIL system is not attributable to random and unobservable factors, again confirming the results of the benchmark regression analysis in this study.

#### 4.3.3 PSM–DID test

To select EPIL pilot areas, the government considers the location and local development characteristics, meaning that the implementation of the EPIL policy was not itself randomly distributed. However, PSM method can help identify control group samples with similar characteristics to the treatment group while satisfying the assumption of balance; then, DID can be used to evaluate the policy effects, thereby reducing endogeneity interference. Therefore, we adopted PSM to match the treatment and control groups and subsequently used staggered DID to verify the influence of EPIL on ULGUE within the matched sample range.

**Fig 3:**
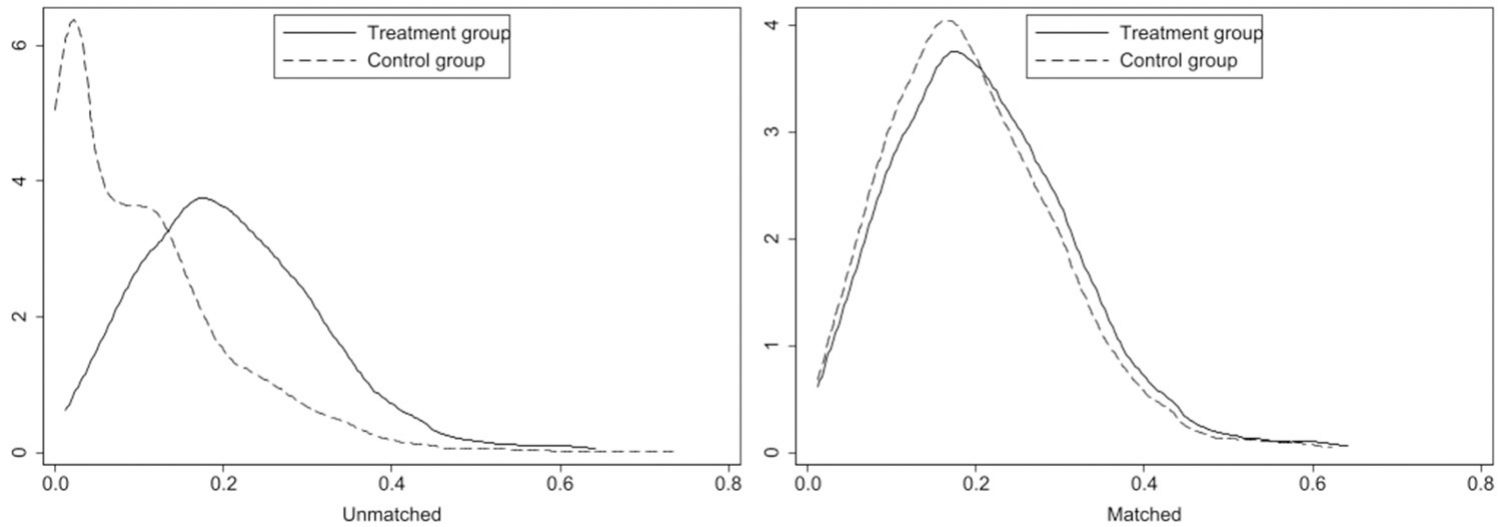
Kernel density plot before and after PSM matching.

Empirically, we matched treatment and control groups based on *GDP* and *GDP*^*2*^ for economic development, *UR*, *LRD*, *FII*, and *URE*. For each treatment form, we obtained one control city with the nearest propensity score without replacement. The balance results following PSM are presented in Table 7. As shown in the table, the standard errors of all covariates post-matching are below 20%, and the results of the t-test fail to reject the null hypothesis, suggesting no systematic difference between the treatment and control groups [46]. Additionally, Fig 3 illustrates that the majority of the propensity scores for the matched samples fall within the same support domain, indicating that the matching method satisfies the common support assumption.

**Table 7:** Balance test following PSM.

*VARIABLES*	Matched/ Unmatched	Mean		Standard bias%	Standard bias reduction %	T-test	
		Treatment group	Control group			t	p>│t│
***GDP***	U	0.06079	0.08978	-79.5	97.5	-12.91	0.000
	M	0.06079	0.06006	2.0		0.34	0.736
***GDP*** ^***2***^	U	0.46726	0.98829	-93.1	95.4	-13.33	0.000
	M	0.46726	0.44353	4.2		1.15	0.251
***UR***	U	0.43834	0.43834	20.3	27.8	3.98	0.000
	M	0.43834	0.40213	14.7		2.00	0.045
***LRD***	U	0.02192	0.0154	37.5	75.0	7.96	0.000
	M	0.02192	0.02029	9.4		1.15	0.249
***FII***	U	0.01415	0.01662	-14.9	78.3	-2.69	0.007
	M	0.01415	0.01468	-3.2		-0.46	0.646
***URE***	U	0.02381	0.05327	-42.0	97.8	-6.29	0.000
	M	0.02381	0.02317	0.9		0.18	0.855

Then, we used the staggered DID model to verify the influence of the EPIL system on ULGUE within the matched sample. The regression results are presented in Columns (3) and (4) of Table 8. Columns (1) and (2) in Table 8 represent the DID regression results prior to PSM; the results show that the estimated coefficients of the EPIL system are significantly positive at 1%, indicating that the implementation of the EPIL policy contributes to the improvement of ULGUE. This provides evidence for the direct impact mechanism of the EPIL on ULGUE, thus confirming our study hypothesis.

**Table 8:** The PSM–DID regression results.

	DID		PSM-DID	
*VARIABLES*	(1)	(2)	(3)	(4)
***policy***_***it***_	0.071***	0.066***	0.068***	0.064***
	(2.90)	(2.76)	(2.80)	(2.75)
***GDP***		0.933***		1.014***
		(4.23)		(4.18)
***GDP*** ^***2***^		-0.051***		-0.049***
		(-3.65)		(-3.18)
***UR***		-0.133		-0.168*
		(-1.28)		(-1.85)
***LRD***		1.725**		0.455
		(2.42)		(0.60)
***FII***		0.432		0.431
		(0.97)		(1.00)
***URE***		0.418**		0.421**
		(2.31)		(2.42)
***Constant***	0.347***	0.312***	0.330***	0.314***
	(120.43)	(6.94)	(144.46)	(7.81)
**City FE**	YES	YES	YES	YES
**Year FE**	YES	YES	YES	YES
**Observations**	3,113	3,113	2,749	2,749
**Adj-R** ^***2***^	0.638	0.647	0.635	0.642

Robust t-statistics in parentheses.

*** p<0.01

** p<0.05

* p<0.1.

#### 4.3.4 Clustered by province

Based on robustness considerations, we also conducted regression analysis at the provincial level for clustering, and the results are reported in Table 9. We found that, consistent with the primary results in Table 5, the coefficients in each column of Table 9 are also significantly positive, further indicating that this study’s conclusion is robust.

**Table 9:** Clusters by province.

*VARIABLES*	(1)	(2)	(3)
***policy***_***it***_	0.197***	0.071*	0.066*
	(6.75)	(1.96)	(1.95)
***GDP***			0.933***
			(3.22)
***GDP*** ^***2***^			-0.051**
			(-2.57)
***Urban***			-0.133
			(-1.17)
***Sr***			1.725*
			(1.88)
***Fi***			0.432
			(0.59)
***Resource***			0.418*
			(2.04)
***Constant***	0.333***	0.753***	0.718***
	(24.45)	(50.74)	(8.63)
**City FE**	YES	YES	YES
**Year FE**	YES	YES	YES
**Observations**	3,113	3,113	3,113
**Adj-R** ^***2***^	0.075	0.638	0.647

Robust t-statistics in parentheses.

*** p<0.01

** p<0.05

* p<0.1.

## 5. Mechanism validation and heterogeneity analysis

### 5.1 Mechanism analysis

The research findings above indicate that the implementation of the EPIL system has significantly improved ULGUE. Next, we explore the underlying mechanisms of this impact. First, the supervisory agencies can not only supervise the enforcement of environmental protection laws, thus playing a role in environmental governance, but also increase the numbers of EPIL plaintiffs, thereby stimulating the public’s enthusiasm to participate in environmental governance. Second, under EPIL, the pilot cities face enhanced environmental constraints and social attention, enabling the local governments to reduce high-pollution and high-energy-consuming industries in their jurisdiction through various policy means. Third, to avoid high penalties for violating environmental regulations, companies are either being forced to comply or are voluntarily engaging in green technological innovation. These transformations in industries and technologies could also be improving ULGUE.

To examine the existence of mechanism channels, following Zhao et al. (2010) we used the mediation effect model for empirical analysis [47]. The model constructs and examines the mediating role of EPIL in promoting the transformation toward green and sustainable development, considering other influential factors such as environmental administrative penalties and public participation. The steps followed for constructing the mediation model in this study are as follows: First, we established the regression model from Eq (1) for the dependent variable and the basic explanatory variables; second, we performed a regression analysis (Eq (4)) for the mediating variables and the basic explanatory variables; finally, a regression analysis (Eq (5)) was conducted for the dependent variable, explanatory variables, and mediating variables simultaneously.


$$Medrator_{it}=\beta_{0}+\beta_{1}policy_{it}+\sum_{j}\alpha_{j}\times control+\gamma_{i}+\theta_{t}+\varepsilon_{it}$$ (4)

$$ULGUE_{it}=\lambda_{0}+\lambda_{1}policy_{it}+\lambda_{2}Medrator_{it}+\sum_{j}\alpha_{j}\times control+\gamma_{i}+\theta_{t}+\varepsilon_{it}$$ (5)

If EPIL significantly influences *ISU*, *lnGTI*, environmental administrative penalties, and public participation in environmental governance, thereby affecting ULGUE in the field of economics, the estimated coefficients *β*₁ and *λ*₂ should both be significant, resulting in a mediating effect of *β*₁ × *λ*₂. Moreover, if the coefficient *λ*₁ in Eq (5) is not significant, the mediation is complete; otherwise, the effect is partial.

#### 5.1.1 Strengthening the construction of the rule of law and enhancing supervision over environmental administrative agencies

According to Table 10, in Column (1) of Eq (4), *policy*_*it*_ is significantly positive at 1%, indicating that implementing EPIL system is significantly increasing the number of environmental administrative penalties and strengthening the efforts of environmental law enforcement agencies in addressing environmental pollution. Moving on to Column (2) in Eq (5), the coefficient of the number of environmental administrative penalties (*lnEES*) is significantly positive at 1%, and the coefficient of the interaction term from bootstrap testing is also significant and positive. This indicates the existence of an indirect effect of the EPIL system. Implementing the policy enables stronger punishment for environmental pollution through environmental civil public interest litigation and promotes effective coordination between judicial and administrative sectors. It creates an optimized path for environmental protection and contributes to improving ULGUE.

**Table 10:** Mechanism analysis results.

*VARIABLES*	Environmental administrative penalty mechanism test		Public participation in environmental protection mechanism test	
	(1)	(2)	(3)	(4)
***Policy***_***it***_	1.228***	0.109***	0.506*	0.064***
	(4.95)	(4.75)	(1.94)	(2.72)
***lnEES***		0.005***		
		(3.11)		
***PPEG***				0.003**
				(2.14)
***GDP***	-8.966**	-0.034	7.192***	0.912***
	(-2.44)	(-0.17)	(2.85)	(4.19)
***GDP*** ^***2***^	-2.918***	-0.040***	0.170	-0.052***
	(-11.73)	(-3.51)	(0.77)	(-3.72)
***UR***	9.059***	0.005	-4.809***	-0.119
	(4.21)	(0.05)	(-3.52)	(-1.16)
***LRD***	45.207***	2.931***	41.076***	1.601**
	(3.25)	(3.89)	(4.02)	(2.28)
***FII***	6.587	-0.213	8.015	0.408
	(0.73)	(-0.44)	(1.12)	(0.92)
***URE***	-15.508***	-0.031	0.699	0.416**
	(-4.65)	(-0.16)	(0.33)	(2.30)
***Constant***	0.827	0.334***	6.075***	0.216***
	(0.83)	(7.59)	(10.25)	(4.37)
**City FE**	YES	YES	YES	YES
**Year FE**	YES	YES	YES	YES
**Observations**	3,113	3,113	3,113	3,113
**Adj-R** ^***2***^	0.606	0.608	0.775	0.262
Bootstrap test	0.0121***		0. 00269***	
	(z = 4.89)		(z = 2.60)	

Robust t-statistics in parentheses.

*** p<0.01

** p<0.05

* p<0.1.

#### 5.1.2 Enhancing the participation of the general public

In examining the role of the general public in environmental governance and establishing external constraints, we observed significantly positive coefficients for the explanatory variable *policy*_*it*_ as shown in Column (3) of Table 10. This implies a significant increase in the number of proposals made by the People’s Congress and the Chinese People’s Political Consultative Conference, reflecting the public’s increasing enthusiasm for participating in environmental protection since EPIL. The communication mechanism between judicial and administrative organs and the general public has been effectively established, thereby reinforcing the external constraints on non-environmental behaviors within society. Moreover, in Column (4), the coefficient of the environmental public participation (*PPEG*) is significantly positive at 5%, and the coefficient cross-term tested by bootstrap is also significant. This indicates that the EPIL system not only enhances public participation in environmental protection but also strengthens external constraints on ecological civilization protection, thereby promoting ULGUE.

#### 5.1.3 Promoting green technology innovation and facilitating the structural transformation and upgrading of industries

Based on these concepts, we examined the indirect effects of industrial structural upgrading (*ISU*) and green technology innovation (*lnGTI*). The regression results are presented in Table 11. In Columns (1) and (2), all coefficients are significant at 1% and positive. The coefficients of the interaction terms in the bootstrap tests are also significant, indicating that industrial structural upgrading acts as a partial mediating variable between the EPIL system and ULGUE. The proportion of the mediating effect to the total effect is 0.174, suggesting that in response to environmental oversight and social pressure, local governments have promoted the transformation and upgrading of industrial structure toward green and low-energy consumption, thereby enhancing ULGUE. One possible explanation is that implementing EPIL promotes the overall upgrading of the industrial chain, providing new impetus for stable growth in industries with high technological content and added value, and resolving the historical trade-off between economic development and environmental protection. Thus, ULGUE improves [21]. Similarly, the results in Column (3) demonstrate that the EPIL system contributes significantly to urban green technology innovation, with a coefficient of 0.441 and statistical significance at 1%. As shown in Column (4), after we incorporated both the core explanatory variable and *lnGTI* into the model, *lnGTI* is significantly positive at 1%. Additionally, the coefficient of the interaction term, tested by bootstrap, is also significantly positive. This finding confirms that the implementation of the EPIL system internalizes the negative externality of environmental pollution into enterprises’ costs through administrative penalties and social public pressure. Consequently, it compels enterprises to engage in green technology innovation to mitigate costs and improve ULGUE.

**Table 11:** Mechanism analysis results.

*VARIABLES*	Industrial Structure Upgrading mechanism test		Green Technology Innovation mechanism test	
	(1)	(2)	(3)	(4)
***policy***_***it***_	0.147***	0.054**	0.441***	0.096***
	(3.84)	(2.30)	(8.70)	(4.18)
***ISU***		0.078***		
		(3.01)		
***lnGTI***				0.041***
				(5.39)
***GDP***	2.751***	0.719***	-2.448***	0.025
	(5.35)	(3.26)	(-2.99)	(0.13)
***GDP*** ^***2***^	-0.045	-0.048***	-0.527***	-0.032***
	(-1.25)	(-3.50)	(-10.07)	(-2.81)
***UR***	0.061	-0.138	1.510***	-0.015
	(0.27)	(-1.36)	(4.78)	(-0.16)
***LRD***	2.102**	1.561**	16.501***	2.463***
	(1.98)	(2.25)	(6.94)	(3.24)
***FII***	-2.912***	0.659	-3.200*	-0.051
	(-2.84)	(1.45)	(-1.89)	(-0.10)
***URE***	2.148***	0.251	-3.357***	0.035
	(5.08)	(1.37)	(-5.67)	(0.19)
***Constant***	0.915***	0.240***	5.096***	0.128**
	(9.07)	(4.75)	(33.38)	(2.25)
**City FE**	YES	YES	YES	YES
**Year FE**	YES	YES	YES	YES
**Observations**	3,113	3,113	3,113	3,113
**Adj-R** ^***2***^	0.855	0.652	0.914	0.613
**Bootstrap test**	0. 0128***		0.00569***	
	(z = 3.42)		(z = 3.57)	

Robust t-statistics in parentheses.

*** p<0.01

** p<0.05

* p<0.1.

### 5.2 Heterogeneity analysis

In the previous analysis, we discussed the overall impact of the EPIL system on ULGUE. However, further analysis is needed to explore the inherent differences among different regions. Prior literature indicates that there are significant differences in regional resources in addition to the economic and social development conditions [48]. These inherent differences likely influence the development goals and assessment indicators that local governments devise, thereby affecting the implementation effects of EPIL. For instance, in cities with highly transparent information disclosures of environmental quality and violations of pollution regulations, local governments face greater pressure from central environmental supervision and public opinion. Consequently, they pay more attention to environmental protection and governance, eliminating obstacles to implementing EPIL pilot work and even providing assistance.

Moreover, the degree of marketization varies among different regions, and the frequency of pollution and other activities that violate market rules differs accordingly. This may also lead to differentiated impacts on the promotion of the pilot work of the EPIL system. Based on these findings, this study will conduct a heterogeneity analysis from the perspectives of urban resource endowment, environmental information disclosure, and marketization level.

#### 5.2.1 City resource endowment

Based on the Sustainable Development Plan for Resource-based Cities (2013−2020) and the classification list of resource-based cities, we classified the total sample of cities as resource-based or not and then conducted a group regression (Table 12). We found a significant positive effect of implementing the EPIL system on the ULGUE of resource-based cities. In contrast, the coefficients of the main explanatory variables in non-resource-based cities were not statistically significant. This could have been because the resource-based cities have abundant mineral resources, resulting in higher industrial energy consumption and pollution levels. The pilot period of the EPIL system, focused on ecological environment and resource protection, and EPIL policy implementation would be likely to promote the green and low-consumption transformation of resource-based cities’ high-pollution and high-energy-consuming industries, thereby improving their ULGUE.

**Table 12:** Analysis of resource endowment heterogeneity.

*VARIABLES*	resource-based city		Non-resource-based city	
	(1)	(2)	(3)	(4)
***Policy***_***it***_	0.098**	0.097**	0.051	0.047
	(2.45)	(2.54)	(1.65)	(1.54)
***GDP***		1.134***		0.890***
		(3.49)		(2.98)
***GDP*** ^***2***^		-0.058***		-0.049**
		(-2.80)		(-2.57)
***UR***		-0.244		-0.108
		(-1.55)		(-0.86)
***LRD***		1.282		1.417
		(1.16)		(1.56)
***FII***		-0.540		0.977*
		(-0.74)		(1.75)
***URE***		0.154		0.139
		(0.78)		(0.35)
***Constant***	0.290***	0.317***	0.385***	0.350***
	(65.89)	(4.69)	(101.31)	(6.22)
**City FE**	YES	YES	YES	YES
**Year FE**	YES	YES	YES	YES
**Observations**	1,232	1,232	1,881	1,881
**Adj-R** ^***2***^	0.533	0.546	0.673	0.679

Robust t-statistics in parentheses.

*** p<0.01

** p<0.05

* p<0.1.

#### 5.2.2 Disclosure of environmental information

In 2008, the Chinese government issued the “Measures for the Disclosure of Environmental Information,” which mandated that environmental protection departments at all levels regularly disclose environmental information. This includes records of regulatory violations by enterprises and the outcomes of public complaints. The purpose of this measure is to enhance transparency in environmental information, strengthen the environmental governance capacity of local governments, and establish a long-term environmental governance mechanism [49].

It was possible that there were differences between regions with disclosed environmental information and those without in the impacts of EPIL on ULGUE. Consequently, we introduced dummy variables based on the list of cities with disclosed environmental information and conducted grouped regression analysis (Table 13); it is evident that the public interest litigation system has a more pronounced impact in regions with disclosed environmental information. Hence, the government should continue to promote transparency in environmental information disclosure and provide informational advantages for the effective implementation of the public interest litigation system.

**Table 13:** Heterogeneity analysis of environmental protection policies.

*VARIABLES*	Environmental information disclosure region		Non-disclosure of environmental information area	
	(1)	(2)	(3)	(4)
***policy***_***it***_	0.099***	0.087***	0.050	0.051
	(2.96)	(2.65)	(1.47)	(1.53)
***GDP***		1.096***		0.828***
		(2.80)		(3.18)
***GDP*** ^***2***^		-0.083***		-0.036**
		(-3.10)		(-2.14)
***UR***		-0.148		-0.121
		(-0.84)		(-1.08)
***LRD***		1.729		1.639*
		(1.62)		(1.87)
***FII***		0.015		0.774
		(0.02)		(1.50)
***URE***		0.039		0.533***
		(0.08)		(2.97)
***Constant***	0.381***	0.395***	0.326***	0.269***
	(88.62)	(4.05)	(85.09)	(6.45)
**City FE**	YES	YES	YES	YES
**Year FE**	YES	YES	YES	YES
**Observations**	1,210	1,210	1,903	1,903
**Adj-R** ^***2***^	0.699	0.709	0.577	0.587

Robust t-statistics in parentheses.

*** p<0.01

** p<0.05

* p<0.1.

#### 5.2.3 Marketization degree

We next used the marketization index proposed by Fan et al. (2001) to measure the level of marketization in different regions [50]. We divided the cities into two categories: high versus low cut off at the median index (Table 14): In cities with high marketization, EPIL can play a significant role in promoting environmental protection, consequently leading to a positive impact on ULGUE. One possible explanation is that in cities with higher levels of marketization, market entities are more inclined to comply with regulations, resulting in more efficient law enforcement and better legal awareness among the general public. This, in turn, strengthens the effectiveness of EPIL policies, ultimately facilitating the improvement of urban ULGUE.

**Table 14:** Analysis of marketization degree heterogeneity.

*VARIABLES*	high degree of marketization		low degree of marketization	
	(1)	(2)	(3)	(4)
***policy***_***it***_	0.088***	0.085***	-0.015	-0.012
	(3.08)	(3.13)	(-0.34)	(-0.28)
***GDP***		0.775**		1.051***
		(2.51)		(4.80)
***GDP*** ^***2***^		-0.077***		-0.047***
		(-2.92)		(-3.23)
***UR***		-0.084		-0.109
		(-0.57)		(-0.77)
***LRD***		2.351**		-0.308
		(2.33)		(-0.31)
***FII***		-0.752		0.688
		(-1.28)		(1.10)
***URE***		0.234		0.150
		(0.73)		(0.82)
***Constant***	0.380***	0.368***	0.315***	0.291***
	(68.36)	(5.62)	(170.02)	(5.14)
**City FE**	YES	YES	YES	YES
**Year FE**	YES	YES	YES	YES
**Observations**	1,529	1,529	1,556	1,556
**Adj-R** ^***2***^	0.730	0.737	0.698	0.704

Robust t-statistics in parentheses.

*** p<0.01

** p<0.05

* p<0.1.

## 6. Conclusion and policy implication

Based on the data from 2010 to 2020 for 283 prefecture-level cities in China, we conducted an empirical examination of the impacts of the country’s environmental public interest litigation on its ULGUE using staggered DID; the research findings show that the pilot of the EPIL policy effectively promotes ULGUE through strengthening environmental supervision and law enforcement, increasing public participation, driving industrial transformation and upgrading, and promoting green technological innovation. EPIL can effectively improve the efficiency of government environmental management regulation enforcement and stimulate the enthusiasm of the public to participate in the process, making it more likely that people will report environmental violations and they can be punished. EPIL also increases the costs to business of land pollution violations, which will stimulate the endogeneity power of green innovation and transformation enterprises based on the balance of cost and benefit. Heterogeneity analysis reveals that the impact of EPIL on ULGUE is more pronounced in resource-based cities, areas with transparent environmental information, and cities with higher degrees of marketization. Based on the above research, we propose the following policy recommendations.

First, importance must be given to procuratorial suggestions and actively participate in public interest litigation. Prosecutorial advice is a statutory prelitigation procedure that must be followed in public interest litigation. When an administrative illegal act is discovered, the procuratorial organ first issues advice to the administrative organ to urge it to perform its duties in accordance with the law. After receiving procuratorial suggestions, administrative agencies should promptly handle and respond, rectify relevant issues, and effectively promote the positive interaction between procuratorial supervision and administrative law enforcement, as well as coordinated governance.

Second, consensus should be gathered on environmental protection and form a joint supervision system for the entire society, strongly promoting ecological and EPIL work. We recommend that administrative organs, judicial organs, procuratorial organs, and news media at all levels must increase their publicity efforts for the public interest litigation system based on the notion that “whoever enforces the law will popularize the law.” It is necessary to provide accurate guidance on handling case information for public release in accordance with the law, improve the social awareness of public interest litigation work, and foster a positive atmosphere for the whole society to jointly supervise the environment and manage public interest litigation.

Third, environmental protection should be promoted, an environmental protection public welfare fund established, and all compensation fees from winning lawsuits to this special fund clearly allocated to repair the environment; pay for identification, litigation, and other fees in public interest litigation work; and reward organizations and individuals who have made outstanding contributions to environmental public welfare litigation or protection industry to mobilize the enthusiasm of the whole society to participate in environmental protection.

Fourth, we should expand EPIL and strengthen the internal power of green innovation to transform and upgrade enterprises via effective public interest litigation and administration. On the one hand, businesses need to establish their own concept of green development and pay attention to the long-term value generated by land green utilization. On the other hand, relevant government departments must pay attention to the difficulties faced by enterprises in the early stage of green innovation and transformation and provide targeted assistance.

Fifth, and finally, developing countries should attach importance to using the rule of law to improve ULGUE. Land is an important factor of economic development, and compared with developed countries, developing countries often face the dual pressures of economic growth and environmental protection more acutely. Achieving green and efficient development and utilization of land is an urgent problem to be solved. In this study, we focused on China, the largest developing country; we determined that strengthening the reform of the environmental justice system can effectively improve ULGUE. Additionally, according to the heterogeneity test results, China’s government should continue to increase the public disclosure of environmental information, strengthen the external market-oriented constraint mechanism, and implement stronger supervision for areas with intensive development of environmental resources such as land to expand the effects of EPIL on ULGUE.

## Supporting information

S1 Data Pilot information of EPIL. (XLS)

## References

[1] Guo B, Lu M, Fan Y, Wu H, Yang Y, Wang C. A novel remote sensing monitoring index of salinization based on three-dimensional feature space model and its application in the Yellow River Delta of China. Geomatics Natural Hazards & Risk. 2023;14(1):95–116. doi: 10.1080/19475705.2022.2156820

[2] Yuan X, Chen L, Sheng X, Li Y, Liu M, Zhang Y, et al. Evaluation of regional sustainability through emergy analysis: a case study of nine cities in the Yellow River Basin of China. Environmental Science and Pollution Research. 2022;29(26):40213–25. doi: 10.1007/s11356-022-18916-6 35119634

[3] Zhang B, Chen X, Guo H. Does central supervision enhance local environmental enforcement? Quasi-experimental evidence from China. Journal of Public Economics. 2018;164:70–90. 10.1016/j.jpubeco.2018.05.009.

[4] Zhai T, Chang Y-C. Standing of environmental public-interest litigants in China: Evolution, obstacles and solutions. Journal of Environmental Law. 2018;30(3):369–97. 10.1093/jel/eqy011.

[5] Tang Y, Wang K, Ji X, Xu H, Xiao Y. Assessment and Spatial-Temporal Evolution Analysis of Urban Land Use Efficiency under Green Development Orientation: Case of the Yangtze River Delta Urban Agglomerations. Land. 2021;10(7):715. doi: 10.3390/land10070715

[6] Yang X, Wu Y, Dang H. Urban Land Use Efficiency and Coordination in China. 2017;9(3):410. doi: 10.3390/su9030410

[7] Tone K. Dealing with undesirable outputs in DEA: a Slacks-Based Measure (SBM) approach. Nippon Opereshonzu, Risachi Gakkai Shunki Kenkyu Happyokai Abusutorakutoshu.2004;2004:44–5. https://www.researchgate.net/publication/284047010.

[8] Zhang L, Yu Y, Chen Y. The spatial-temporal evolution characteristics and driving factors of the green utilization efficiency of urban land in China. Frontiers in Environmental Science. 2022;10:955982. 10.1111/grow.12465.

[9] Chai Z, Guo F, Cao J, Yang X. The road to eco-efficiency: Can ecological civilization pilot zone be useful? New evidence from China. Journal of Environmental Planning and Management. 2022:1–27. 10.1080/09640568.2022.2118571.

[10] Hanif I. Impact of economic growth, nonrenewable and renewable energy consumption, and urbanization on carbon emissions in Sub-Saharan Africa. Environmental Science and Pollution Research. 2018;25(15):15057–67. doi: 10.1007/s11356-018-1753-4 29552722

[11] Chakraborty P, Chatterjee C. Does environmental regulation indirectly induce upstream innovation? New evidence from India. Research Policy. 2017;46(5):939–55. 10.1016/j.respol.2017.03.004.

[12] Albrizio S, Kozluk T, Zipperer V. Environmental policies and productivity growth: Evidence across industries and firms. Journal of Environmental Economics and Management. 2017;81:209–26. 10.1016/j.jeem.2016.06.002.

[13] Wang A, Lin W, Liu B, Wang H, Xu H. Does Smart City Construction Improve the Green Utilization Efficiency of Urban Land? 2021;10(6):657. doi: 10.3390/land10060657

[14] Hille E, Möbius P. Environmental Policy, Innovation, and Productivity Growth: Controlling the Effects of Regulation and Endogeneity. Environmental and Resource Economics. 2019;73(4):1315–55. doi: 10.1007/s10640-018-0300-6

[15] Gao Q, Whittaker S. Standing to sue beyond individual rights: who should Be eligible to bring environmental public interest litigation in China? Transnational Environmental Law. 2019;8(2):327–47. 10.3390/su9030410.

[16] Cooper J. Public interest law revisited. Commonwealth Law Bulletin. 1999;25(1):135–53. 10.1080/03050718.1999.9986531.

[17] Liu W.; Fan W Y., Does Public Interest Litigation Promote the Performance of Urban Environmental Governance? An Empirical Study Based on Micro-data of 287 Prefecture-level Cities. Journal of Shanghai University of Finance and Economics 2021; 23(04): 48–62.

[18] Liu Y., The Judicial Practice and Theoretical Exploration of Public Interest Litigation Brought by the Procuratorate. Journal of National Prosecutors College 2017; 25(02): 3–18+170.

[19] Jiang H, Blazey P, Wang Y, Ashiabor H. China’s new approach to environmental governance and environmental public interest litigation. Asia Pacific Journal of Environmental Law. 2020;23(1):39–73. 10.4337/apjel.2020.01.02.

[20] Zhuang H, Wolf SA. Environmental public interest litigation: new roles for civil society organizations in environmental governance in China. Environmental Sociology. 2021;7(4):393–406. 10.1080/23251042.2021.1897243.

[21] Zhang X, Lu X, Chen D, Zhang C, Ge K, Kuang B, et al. Is environmental regulation a blessing or a curse for China’s urban land use efficiency? Evidence from a threshold effect model. Growth and Change. 2021;52(1):265–82. 10.1111/grow.12465.

[22] Zhang J.; Fan W.; Gao Y., Environmental Judicial System Reform and Local Green Innovation: Evidence from Pilot Public Interest Litigation. Journal of Finance and Economics. 2022;48(10): 19–33.

[23] Chen T.; Shao J.; Wang X., Testing the effectiveness and improvement path of the administrative public interest litigation system: An empirical analysis based on the double difference metho. Peking University Law Journal. 2020; 32(05):1328–1352.

[24] Ge T, Hao X, Li J. Effects of public participation on environmental governance in China: A spatial Durbin econometric analysis. Journal of Cleaner Production. 2021;321:129042. 10.1016/j.jclepro.2021.129042

[25] Zhang H, Xu T, Feng C. Does public participation promote environmental efficiency? Evidence from a quasi-natural experiment of environmental information disclosure in China. Energy Economics 2022;108:105871. 10.1016/j.eneco.2022.105871

[26] Almer C, Goeschl T. Environmental Crime and Punishment: Empirical Evidence from the German Penal Code. Land Economics. 2010;86(4):707–726. 10.3368/le.86.4.707

[27] Edwards V. A Review of the Court of Justice’s Case Law in Relation to Waste and Environmental Impact Assessment: 1992–2011. Journal of Environmental Law. 2013;25(3):515–530. 10.1093/jel/eqt026.

[28] Zhang Q, Yu Z, Kong D. The real effect of legal institutions: Environmental courts and firm environmental protection expenditure. Journal of Environmental Economics and Management. 2019;98. 10.1016/j.jeem.2019.102254.

[29] Porter ME, van der Linde C. Toward a New Conception of the Environment-Competitiveness Relationship Journal of Economic Perspectives. 1995;9(4):97–118. https://www.aeaweb.org/articles?id=10.1257/jep.9.4.97.

[30] Horbach J, Rennings K. Environmental innovation and employment dynamics in different technology fields–an analysis based on the German Community Innovation Survey 2009. Journal of Cleaner Production 2013;57:158–65. 10.1016/j.jclepro.2013.05.034.

[31] Du K, Cheng Y, Yao X. Environmental regulation, green technology innovation, and industrial structure upgrading: The road to the green transformation of Chinese cities. Energy Economics. 2021;98:105247. 10.1016/j.eneco.2021. 105247.

[32] Cheng Z, Li X. Do raising environmental costs promote industrial green growth? A Quasi-natural experiment based on the policy of raising standard sewage charges. Journal of Cleaner Production. 2022;343:131004. 10.1016/j.jclepro. 2022.%20131004.

[33] Dong Y, Jin G, Deng X. Dynamic interactive effects of urban land-use efficiency, industrial transformation, and carbon emissions. Journal of Cleaner Production. 2020;270:122547. 10.1016/j.jclepro.2019.117848.

[34] Zhang G, Zhang P, Zhang ZG, Li J. Impact of environmental regulations on industrial structure upgrading: An empirical study on Beijing-Tianjin-Hebei region in China. Journal of Cleaner Production. 2019;238:117848. 10.1016/j.jclepro.2020.122547.

[35] Beck T, Levine R, Levkov A. Big bad banks? The winners and losers from bank deregulation in the United States. The journal of finance. 2010;65(5):1637–67. 10.1111/j.1540-6261.2010.01589.x.

[36] Feng Y, Li Y, Nie C. The Effect of Place-Based Policy on Urban Land Green Use Efficiency: Evidence from the Pilot Free-Trade Zone Establishment in China. Land. 2023;12(3):701. doi: 10.3390/land12030701

[37] Tan S, Hu B, Kuang B, Zhou M. Regional differences and dynamic evolution of urban land green use efficiency within the Yangtze River Delta, China. Land Use Policy. 2021;106:105449. 10.1016/j.landusepol.2021.105449.

[38] Tone K. A slacks-based measure of super-efficiency in data envelopment analysis. European Journal of Operational Research. 2002;143(1):32–41. 10.1016/S0377-2217(01)00324-1.

[39] Wang Z, Jia H, Xu T, Xu C. Manufacturing industrial structure and pollutant emission: An empirical study of China. Journal of Cleaner Production. 2018;197:462–71. 10.1016/j.jclepro.2018.06.092.

[40] Kuang B, Liu J, Fan X. Has China’s Low-Carbon City Construction Enhanced the Green Utilization Efficiency of Urban Land? International Journal of Environmental Research and Public Health. 2022;19(16),9844. doi: 10.3390/ijerph19169844 36011480 PMC9407921

[41] Du W, Li M, Fan Y, Liang S. Can public environmental concern inhibit the market entry of polluting firms: Micro evidence from China. Ecological Indicators. 2023;154. 10.1016/j.ecolind.2023.110528.

[42] Ho K-C, Yan C, Gozgor G, Gu Y. Energy related public environmental concerns and intra-firm pay gap in polluting enterprises: Evidence from China. Energy Economics. 2024;130. 10.1016/j.eneco.2024.107320.

[43] Yu C, Long H, Zhang X, Tan Y, Zhou Y, Zang C, et al. The interaction effect between public environmental concern and air pollution: Evidence from China. Journal of Cleaner Production. 2023;391. 10.1016/j.jclepro.2023.136231.

[44] Zhou Q, Zhu M, Qiao Y, Zhang X, Chen J. Achieving resilience through smart cities? Evidence from China. Habitat International. 2021;111:102348. 10.1016/j.habitatint.2021.102348.

[45] Lu Y, Tao Z, Zhu L. Identifying FDI spillovers. Journal of International Economics. 2017;107:75–90. 10.1016/j.jinteco.2017.01.006.

[46] Rosenbaum PR, Rubin DB. The central role of the propensity score in observational studies for causal effects. Biometrika. 1983;70(1):41–55. 10.1093/biomet/70.1.41.

[47] Zhao X, Lynch J, Chen Q. Reconsidering Baron and Kenny: Myths and truths about mediation analysis. Journal of consumer research. 2010;37(2):197–206. 10.1086/651257.

[48] Li S, Wang D, Wu QJFiES. Effect of ecological civilization pilot demonstration area construction on urban land green use efficiency. Frontiers in Environmental Science. 2023;11:1200171. 10.3389/fenvs.2023.1200171.

[49] Pien C-p. Local environmental information disclosure and environmental non-governmental organizations in Chinese prefecture-level cities. Journal of Environmental Management. 2020; 275: 111225. doi: 10.1016/j.jenvman.2020.111225 32889416

[50] Fan G.; Wang X L.; Zhang L W., Marketization Index for China’s Provinces. Economic Research Journal. 2001; (06): 58–61.

